# Fast generation of lung SBRT plans with a knowledge‐based planning model on ring‐mounted Halcyon Linac

**DOI:** 10.1002/acm2.13427

**Published:** 2021-09-25

**Authors:** Justin Visak, Aaron Webster, Mark E. Bernard, Mahesh Kudrimoti, Marcus E. Randall, Ronald C. McGarry, Damodar Pokhrel

**Affiliations:** ^1^ Medical Physics Graduate Program Department of Radiation Medicine University of Kentucky Lexington Kentucky USA

**Keywords:** coplanar geometry, Halcyon Linac, KBP model, lung SBRT, non‐coplanar VMAT

## Abstract

**Purpose:**

To demonstrate fast treatment planning feasibility of stereotactic body radiation therapy (SBRT) for centrally located lung tumors on Halcyon Linac via a previously validated knowledge‐based planning (KBP) model to support offline adaptive radiotherapy.

**Materials/methods:**

Twenty previously treated non‐coplanar volumetric‐modulated arc therapy (VMAT) lung SBRT plans (*c*‐Truebeam) on SBRT‐dedicated C‐arm Truebeam Linac were selected. Patients received 50 Gy in five fractions. *c*‐Truebeam plans were re‐optimized for Halcyon manually (*m*‐Halcyon) and with KBP model (*k*‐Halcyon). Both *m*‐Halcyon and *k*‐Halcyon plans were normalized for identical or better target coverage than clinical *c*‐Truebeam plans and compared for target conformity, dose heterogeneity, dose fall‐off, and dose tolerances to the organs‐at‐risk (OAR). Treatment delivery parameters and planning times were evaluated.

**Results:**

*k*‐Halcyon plans were dosimetrically similar or better than *m*‐Halcyon and *c*‐Truebeam plans. *k*‐Halcyon and *m*‐Halcyon plan comparisons are presented with respect to *c*‐Truebeam. Differences in conformity index were statistically insignificant in *k*‐Halcyon and on average 0.02 higher (*p* = 0.04) in *m*‐Halcyon plans. Gradient index was on average 0.43 (*p* = 0.006) lower and 0.27 (*p* = 0.02) higher for *k*‐Halcyon and *m*‐Halcyon, respectively. Maximal dose 2 cm away in any direction from target was statistically insignificant. *k*‐Halcyon increased maximal target dose on average by 2.9 Gy (*p* < 0.001). Mean lung dose was on average reduced by 0.10 Gy (*p* = 0.004) in *k*‐Halcyon and increased by 0.14 Gy (*p* < 0.001) in *m*‐Halcyon plans. *k*‐Halcyon plans lowered bronchial tree dose on average by 1.2 Gy. Beam‐on‐time (BOT) was increased by 2.85 and 1.67 min, on average for *k*‐Halcyon and *m*‐Halcyon, respectively. *k*‐Halcyon plans were generated in under 30 min compared to estimated dedicated 180 ± 30 min for *m*‐Halcyon or *c*‐Truebeam plan.

**Conclusion:**

*k*‐Halcyon plans were generated in under 30 min with excellent plan quality. This adaptable KBP model supports high‐volume clinics in the expansion or transfer of lung SBRT patients to Halcyon.

## 1 INTRODUCTION

Surgical resection is an important treatment for early‐stage nonsmall‐cell lung cancer (NSCLC) patients. However, many patients are inoperable due to comorbidities, refuse surgical resection, or present with a high chance of post‐operative morbidity.^1^, ^2^ For these NSCLC patients, stereotactic body radiation therapy (SBRT) has become an extremely effective curative treatment modality.^2^ Compared to poor tumor local‐control rates from conventional lung radiotherapy (60%–70% local failure rates), lung SBRT has provided very high local‐control rates up to 97% (median, 3 years actuarial) with less treatment‐related toxicity compared to surgery.^1^, ^2^, ^3^, ^4^ To deliver high‐quality lung SBRT treatments, a precise delivered dose must be highly conformal around the tumor with a steep dose gradient to limit intermediate dose spillage.^5^ This can be accomplished using traditional 3D conformal radiation therapy (3D‐CRT), intensity‐modulated radiation therapy (IMRT), or more recently via manually generated volumetric‐modulated arc therapy (VMAT) plans.^6^, ^7^, ^8^ Delivering lung SBRT with VMAT provides enhanced dosimetric benefits and faster treatments that may aid in patient compliance.^7^, ^8^ Currently, VMAT lung SBRT treatment is being delivered with flattening filter‐free (FFF) beams using an SBRT‐dedicated C‐arm linac.^2^, ^9^, ^10^, ^11^ FFF beams provide significantly higher dose rates, less out‐of‐field scatter dose, less electron contamination, and better target coverage at the tumor–lung interface in comparison to flattened beams.^11^ These additional benefits translate to superior treatment in a shorter overall treatment time.

**FIGURE 1:**
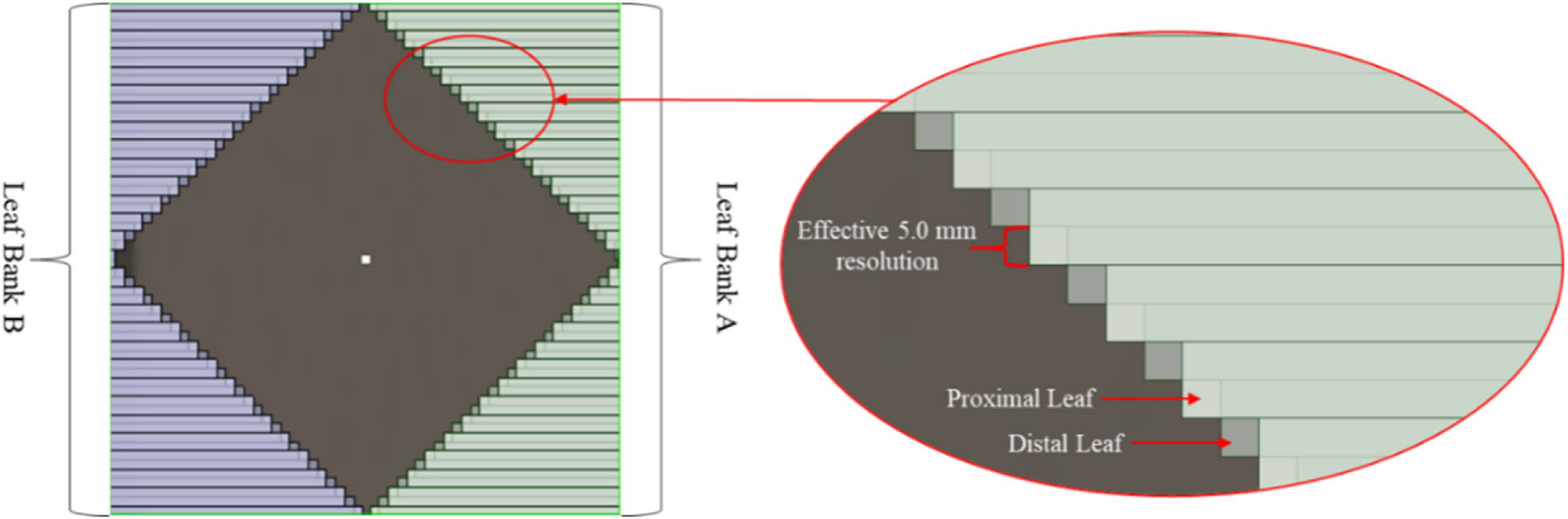
Beams‐eye‐view and description of the new stacked and staggered MLC design on the Halcyon Linac

For fast patient throughput and the advancement of standard radiation treatments to the under‐served communities, Varian Medical Systems (Palo Alto, CA) recently introduced a new jawless, single energy, and ring‐mounted coplanar restricted Halcyon (V2.0) medical linear accelerator.^12^ The Halcyon Linac is equipped with a 6X‐FFF beam with a maximum dose rate setting of up to 800 MU/min, much lower than the 6MV‐FFF (up to 1400 MU/min) beam on an SBRT‐dedicated Truebeam Linac. The Halcyon Linac has a relatively softer beam with a mean energy and nominal maximum depth dose of 1.3 MeV and at 1.3 cm, compared to the corresponding 6MV‐FFF beam on Truebeam of 1.4 MeV and 1.5 cm. Additionally, Halcyon's ring‐mounted gantry design offers a fourfold increase in gantry rotation speed when compared to Truebeam and is equipped with a new design of 1 cm wide dual‐layered stacked and staggered multileaves collimator (MLC) (see Figure 1).^13^, ^14^ This design restricts the field size to 28 × 28 cm^2^; however, the stacked and staggered MLC design offers complete leaf interdigitation and allows for MLC travel all the way to 28 cm. With a less rounded MLC leaf design, the Halcyon boasts a small dosimetric leaf gap of 0.1 mm and ultra‐low leakage and transmission around 0.4%.^14^, ^15^ This novel MLC design offers leaf speed of up to 5.0 cm/s with an effective equivalent 5 mm MLC resolution at the treatment isocenter. Additionally, target localization is potentially improved with the Halcyon onboard imager because it has an advanced image reconstruction algorithm that can iteratively reconstruct a pre‐treatment conebeam CT.^16^ This reconstruction can be acquired in less than 15 s due to the increased gantry speed. Moreover, the new “one‐step patient set up” approach includes automatically applied isocenter shifts that will significantly reduce patient set up times.^12^ One drawback to the Halcyon is that all treatment plans are restricted to coplanar beam geometry, whereas SBRT‐dedicated C‐arm linacs allow for larger range of non‐coplanarity.

Currently, highly conformal clinical VMAT lung SBRT plans are generated using a manually optimized inverse planning technique. An issue with manual planning is that the quality of the final plan depends on individual patient anatomy, planning experience, and available treatment planning time.^17^, ^18^, ^19^, ^20^, ^21^, ^22^, ^23^, ^24^ This potentially leads to interplanner variability (i.e., plans with varied dosimetric quality). Knowledge‐based planning (KBP) has become a clinically feasible approach for generating high‐quality treatment plans and aims to mitigate the issues associated with manual planning by standardizing treatment plans and removing interplanner variability.^18^ This is commonly accomplished by using a model library of previously generated high‐quality clinical plans to predict new treatment parameters.^19^ KBP improves plan quality and drastically reduces treatment planning and will shorten ‘simulation‐to‐treatment’ time down to as few as three working days.^25^ Our center uses Varian RapidPlan dose–volume histogram estimation algorithm as our KBP engine. In the past, a few investigators have shown that KBP may help to create dosimetrically superior or similar lung SBRT plans when compared to manual planning for traditional SBRT‐dedicated C‐arm linac treatments.^21^, ^22^, ^23^, ^24^, ^25^

Halcyon has been shown to provide fast and effective treatment in the setting of conventionally fractionated cranial, head and neck, prostate, and breast treatments.^26^, ^27^, ^28^ However, due to the lack of lung SBRT training datasets on Halcyon, there is no literature that describes training and clinically validating a KBP model for lung SBRT. This prompted us to evaluate the feasibility of generating lung SBRT plans for centrally located tumors as per RTOG‐0813 criteria^29^ on the Halcyon Linac using a previously validated KBP model using high‐quality non‐coplanar Truebeam VMAT plans. It has previously been demonstrated that lung SBRT using a coplanar geometry produces similar patient outcomes compared to non‐coplanar treatments and that it is feasible to treat lung SBRT on the Halcyon Linac.^30^, ^31^ The aim of this study was to evaluate the capabilities of KBP modeling techniques to produce coplanar VMAT plans of similar or better quality on ring‐mounted Halcyon when compared to traditional non‐coplanar lung SBRT treatments delivered on a C‐arm Truebeam. We additionally sought to demonstrate whether the Halcyon can overcome coplanar restrictions with the aid of a previously trained KBP model in the treatment of centrally located lung tumors using SBRT.

## 2 MATERIALS AND METHODS

### 2.1 Patient selection

Institutional review board approval was obtained to conduct this retrospective study. The previously validated in‐house lung SBRT KBP model that was built using highly conformal non‐coplanar VMAT plans with a patient cohort of 86 patients was adopted for this study. Details of the model generation have been published.^25^ Twenty new patients who were previously treated to 50 Gy in five fractions for early‐stage I‐II NSCLC on SBRT‐dedicated Truebeam Linac using non‐coplanar VMAT plans were retrospectively selected to further validate this model on Halcyon Linac.

### 2.2 Clinical plans (*c*‐Truebeam)

Patients in this cohort were primarily immobilized using the Body Pro‐Lock system (CIVCO system, Orange City, IA, USA) in the supine position with their arms above the head and abdominal compression. A free‐breathing 3DCT scan was then performed and a gross target volume (GTV) was delineated followed by a planning target volume (PTV) with expanded margins of 1.0 cm superior/inferior and 0.5 cm laterally from the GTV. If a patient was unable to undergo abdominal compression, a respiration‐correlated 4DCT scan using the Varian RPM system (version 1.7) was performed. Maximum intensity projection (MIP) images were derived from the 4DCT scan, and the images were co‐registered to the free‐breathing 3DCT images to delineate an internal target volume (ITV), therefore, the GTV = ITV. The PTV was created by expanding the ITV by 0.5 cm in all directions per SBRT protocol guidelines. For these patients, all treatment planning was performed on the free‐breathing CT dataset. As specified by the RTOG‐0813 requirements, all relevant organs‐at‐risk (OAR) were contoured (e.g., total lungs, spinal cord, ribs, heart, esophagus brachial plexus, and skin). For the robust validations of this model, we have included variable tumor sizes and locations on both lungs’ geometries as shown in Table 1.

**TABLE 1:** Patient cohort and tumor characteristics

Tumor location	Population, *n*	GTV size (cc)	PTV size (cc)
Overall patient cohort	20	9.7 ± 13.4 (0.1–61.2)	32.6 ± 25.8 (7.5–114.3)
RLL	6	13.5 ± 21.7 (0.1–61.2)	37.0 ± 36.9 (7.5–114.3)
RUL	5	12.8 ± 8.1 (1.6–22.1)	43.1 ± 22.1 (11.2–71.7)
LLL	4	4.2 ± 1.7 (2.8–7.0)	24.1 ± 8.6 (12–33.1)
LUL	5	6.6 ± 5.5 (1.0–14.8)	23.7 ± 14.5 (9.7–51.3)

*Note*: Overall, the patient cohort and each tumor geographical location and tumor sizes are presented as a total number (*n*) and mean ± SD (range), respectively.

Abbreviations: GTV, gross tumor volume; LLL, left lower lobe; LUL, left upper lobe; PTV, planning target volume; RLL, right lower lobe; RUL, right upper lobe.

All patients were treated with a highly conformal plan using non‐coplanar VMAT geometry on Truebeam Linac using a 6MV‐FFF beam with a maximum dose rate of 1400 MU/min. On average three to six non‐coplanar partial arcs (±5–12° couch rotations) were utilized with average arc length of approximately 200° and patient‐specific collimator angles were selected to minimize the MLC tongue and groove effect (jaw‐tracking enabled). Truebeam couch and SBRT board were inserted. Isocenter was placed in the center of the PTV and the dose was prescribed to the 60%–80% isodose line and normalized to ensure at least 95% of the PTV received the full prescription dose of 50 Gy in five fractions. All hot‐spots (average: 120%–140%) were constrained to be within the GTV. Clinical plans were inversely optimized using the photon optimizer (PO version 13.0 or 15.0) with final dose calculation performed using AcurosXB with a 1.25 mm calculation‐grid size (CGS) with tissue heterogeneity corrections.^32^, ^33^ Dose to medium reporting mode was enabled per our linac calibration. These patients were treated every other day. On the treatment day, an online pretreatment cone‐beam CT scan was performed for patient set‐up corrections.

### 2.3 *m‐*Halcyon plans

For comparison, all *c*‐Truebeam lung SBRT plans were manually reoptimized on Halcyon (*m*‐Halcyon) with identical arc lengths and collimator rotations but using coplanar geometry. Additionally, in most cases the total number of arcs were identical to *c*‐Truebeam plans. The Truebeam couch was removed and the Halcyon couch and SBRT board were inserted. As previously described, *m*‐Halcyon plans were reoptimized with the same calculation algorithms (with corresponding CGS) and identical planning objectives when compared to *c*‐Truebeam plans. No jaw‐tracking option is available on this jawless Halcyon. The *m*‐Halcyon plans received the same target coverage as the clinical *c*‐Truebeam plans.

### 2.4 *k‐*Halcyon plans

*c*‐Truebeam plans were reoptimized automatically on Halcyon (*k*‐Halcyon) using a previously validated KBP RapidPlan model. The previous KBP model was developed using 86 high‐quality non‐coplanar VMAT training plans that were previously treated on the Truebeam Linac. All these patients received 50 or 55 Gy in five fractions and treatment was delivered every other day. Additionally, 20 new independent patient's plans were used for KBP model validation. At the time of this manuscript preparation, this model has been deployed clinically on a limited basis. The planning geometry of the *k*‐Halcyon is identical to *m*‐Halcyon. *k*‐Halcyon plans used the same calculation algorithms and corresponding CGS but with the automatic planning constraints generated by the previously validated non‐coplanar KBP model. The *k*‐Halcyon plans received the same target coverage as the clinical *m*‐Halcyon plans.

### 2.5 Plan dosimetric evaluation

All plans were dosimetrically evaluated for target conformity, gradient indices, and dose to OAR with RTOG‐0813 protocol's requirements. This included the ratio of prescription isodose volume to the PTV volume, conformity index (CI), and the ratio of the 50% isodose volume to the PTV volume known as the gradient index (GI). Additionally, the maximal dose at 2 cm away from the PTV in any direction (D2cm) was assessed for intermediate dose fall‐off. Supplemental to the RTOG‐0813 criteria, our institution records the gradient distance (GD) defined as the average distance between the 100% and 50% isodose line to further quantify intermediate dose spillage. Moreover, the heterogeneity index (HI), ratio of PTV maximal dose in cGy and prescription dose were used to assess hot‐spots of each plan. In addition to target and plan complexity metrics, dose to OAR was tracked and documented for maximal and volumetric dosing per RTOG‐0813 criteria. These structures include: spinal cord, ipsilateral brachial plexus, skin, total lung‐PTV, esophagus, heart, and trachea. Plan complexity was simply assessed by recording total number of monitor units (MU) and modulation factor (MF). The MF is defined as the total number of MU divided by the prescription dose in cGy and the corresponding beam‐on‐time (BOT) was calculated using total MU divided by the delivered dose rate for each plan. Moreover, overall treatment planning time and results of independent dose verification via a second physics check Monte Carlo (MC) routine were recorded.^34^ To collect and statistically compare these metrics, an in‐house data collection routine was developed using Eclipse Visual Scripting (Varian Medical Systems, Palo Alto, CA, USA) and MATLAB (Math Works, Natick, MA, USA). Statistical analysis was performed with Microsoft Excel (Microsoft Corp., Redmond, WA, USA) using a paired student *t*‐test with *p* < 0.05 signifying statistical significance.

## 3 RESULTS

### 3.1 Target coverage and intermediate dose fall‐off

Plan quality and target coverage indices are displayed in Table 2. All results are presented for both *m‐*Halcyon and *k‐*Halcyon plans with respect to *c‐*Truebeam plans. Both *m‐*Halcyon and *k‐*Halcyon produced clinically insignificant differences in conformity indices indicating similar target coverage. The *m‐*Halcyon plans, on average, produced less homogenous plans as indicated by the increase of 0.27 in GI (*p* = 0.02), whereas *k‐*Halcyon plans provided more homogeneity in target coverage by reducing the GI on average 0.43 (*p* < 0.005). Across both plans, D2cm differences were statically insignificant; however, the GD was much higher in *m‐*Halcyon plans and lower in *k‐*Halcyon plans. This potentially indicates less intermediate dose spillage when using KBP with Halcyon Linac.

**TABLE 2:** Evaluation of plan quality and target coverage indices for all 20 lung stereotactic body radiation therapy (SBRT) validation cases including original *c*‐Truebeam plans

Target	Parameter	*k*‐Halcyon	*m*‐Halcyon	*c*‐Truebeam	*k*‐Halcyon vs. *m‐*Halcyon, *p* value	*k*‐Halcyon vs. *c*‐Truebeam, *p* value	*m*‐Halcyon vs. *c‐*Truebeam, *p* value
PTV	CI	1.01 ± 0.02	1.03 ± 0.04	1.01 ± 0.04	n.s.	n.s.	**0.04**
	HI	1.19 ± 0.02	1.16 ± 0.03	1.14 ± 0.04	**<0.001**	**<0.001**	n.s.
	GI	4.22 ± 1.2	4.92 ± 1.3	4.65 ± 1.2	**<0.001**	**0.005**	**0.02**
	D2cm (%)	50.8 ± 4.9	51.4 ± 5.0	50.5 ± 4.4	n.s.	n.s.	n.s.
	GD (cm)	1.06 ± 0.2	1.17 ± 0.2	1.11 ± 0.2	**<0.001**	**0.02**	**0.002**

Note: Mean ± SD and *p*‐values were reported. Significant *p*‐values are highlighted in bold.

Abbreviations: CI, conformity index; D2cm, the maximal dose at 2 cm away from the PTV in any direction; GD, gradient distance; GI, gradient index; HI, heterogeneity index; n.s., not significant; PTV, planning target volume.

**FIGURE 2:**
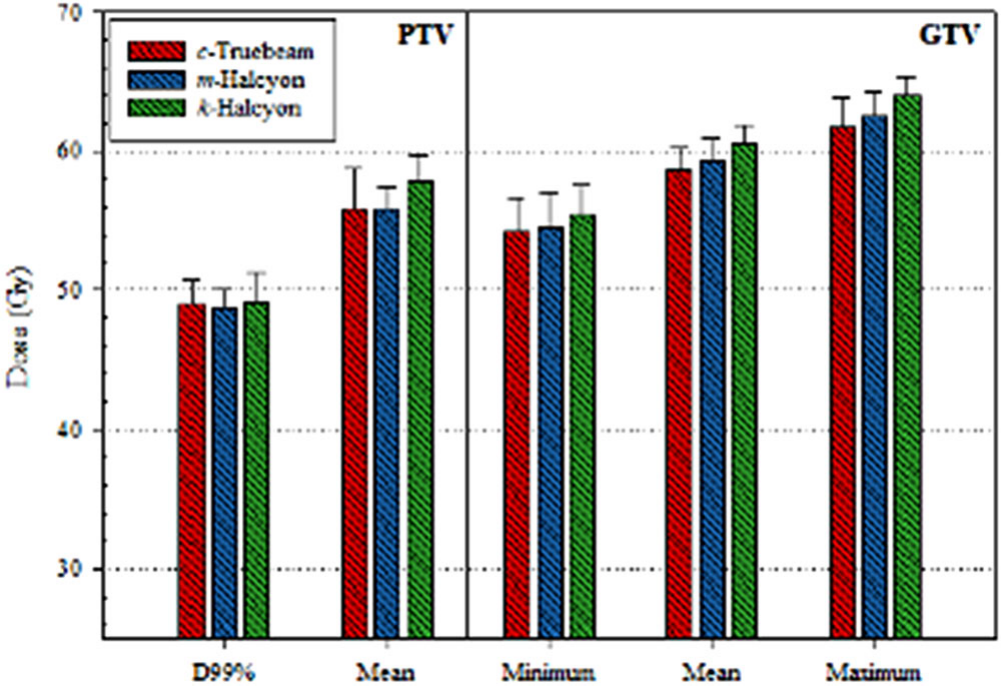
Average doses to planning target volume (PTV) and gross tumor volume (GTV) (in Gy) for 20 lung stereotactic body radiation therapy (SBRT) validation cases including clinically treated *c‐*Truebeam plans. Near minimum PTV (D99%) dose was similar across *c‐*Truebeam, *m‐*Halcyon, and *k‐*Halcyon plans. In general, *k‐*Halcyon provided higher average dose to PTV and GTV relative to both *m‐*Halcyon and *k‐*Halcyon plans

Figure 2 displays the average near minimum (D99%) and mean PTV dose including the average minimum, mean, and maximal doses to GTV for all three plans. Qualitatively, the near minimum dose to PTV was similar across all three plans (*p* = n.s.), however *k‐*Halcyon was able to increase dose across all other metrics indicating slight dose escalation may be achievable on the Halcyon using KBP model. This is in part due to selected target dosing objectives chosen by the KBP model and to the coplanarity treatments on Halcyon plans. The PTV mean dose was 55.8 ± 1.61 Gy (53.7–60.6 Gy), 57.8 ± 1.81 Gy (55.7–65.0 Gy), and 55.7 ± 3.0 Gy (53.9–68.3 Gy) for *m‐*Halcyon, *k‐*Halcyon, and *c‐*Truebeam plans, respectively. Additionally, on average *k‐*Halcyon plans provided higher GTV minimum dose by 1.2 Gy (*p* = 0.04), maximum up to 7.6 Gy in some cases.

### 3.2 Dose to OAR

**FIGURE 3:**
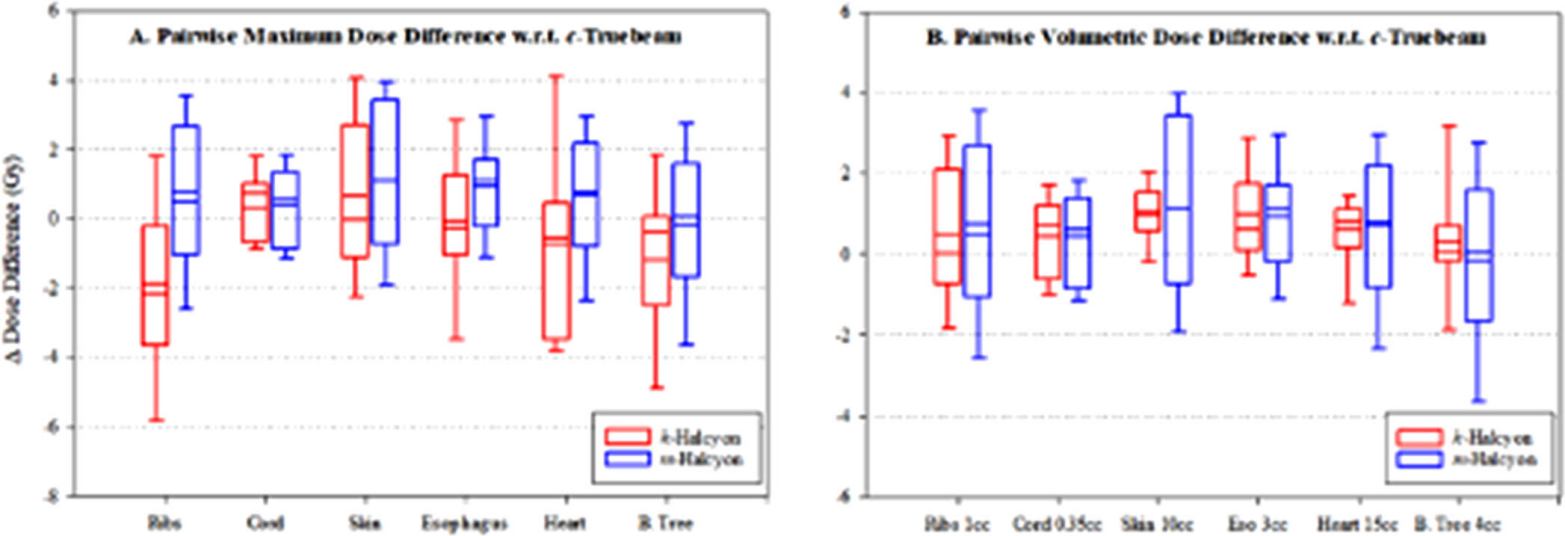
Pairwise dose differences (in Gy) of maximal and volumetric dose to organs‐at‐risk (OAR) for *k‐*Halcyon and *m‐*Halcyon plans with respect to *c‐*Truebeam plans. Positive values indicate respective plans on average provided a higher dose to OAR compared to *c‐*Truebeam plans. *k‐*Halcyon plans across many dosing metrics delivered lower OAR doses than *m‐*Halcyon including an average 0.74 Gy reduction in maximal dose to heart

Maximal and volumetric doses to OAR were recorded per protocol guidelines and the pairwise differences (in Gy) with respect to clinical *c‐*Truebeam plans are presented in Figure 3. Positive values indicate that both *m‐*Halcyon and *k‐*Halcyon plans provided higher doses to OAR compared to *c‐*Truebeam plans. In many cases, *k‐*Halcyon plans helped reduce doses to OAR more than *m‐*Halcyon plans, although these small differences may not be clinically significant. However, this finding supports the premise that *k‐*Halcyon plans on average will be dosimetrically similar or superior to clinical *c‐*Truebeam plans and to *m‐*Halcyon. One interesting value to note is that *k‐*Halcyon on average reduced the maximum rib dose by 1.9 Gy (*p* = 0.003), maximum up to 6.3 Gy in some cases.

Dose to normal lung was evaluated using V5Gy, V10Gy, V20Gy and mean lung dose (Gy) as all of these metrics have been correlated to radiation‐induced pneumonitis and recently, may correlate with overall survival in stage I lung cancer.^35^, ^36^, ^37^ These results are shown in Table 3. *k‐*Halcyon plans on average reduced all normal lung dosing metrics whereas *m‐*Halcyon increased values when compared to *c‐*Truebeam plans. The largest differences in dose to normal lung were recorded for V5Gy. There was a 0.4% decrease and a 0.6% increase of dose for *k‐*Halcyon and *m‐*Halcyon, respectively.

**TABLE 3:** Evaluation of normal lung dosing for 20 lung stereotactic body radiation therapy (SBRT) validation cases including original *c*‐Truebeam plans

Lungs‐PTV	*k*‐Halcyon	*m*‐Halcyon	*c*‐Truebeam	*k*‐Halcyon vs. *m*‐Halcyon, *p* value	*k*‐Halcyon vs. *c*‐Truebeam, *p* value	*m*‐Halcyon vs. *c*‐Truebeam, *p* value
V5Gy (%)	12.0 ± 5.6	13 ± 5.6	12.4 ± 5.6	**<0.001**	n.s.	**<0.001**
V10Gy (%)	6.9 ± 4.2	7.6 ± 4.4	7.2 ± 4.3	**<0.001**	**0.01**	**<0.001**
V20Gy (%)	2.7 ± 2.1	3.0 ± 2.3	2.8 ± 2.2	**<0.001**	**0.002**	**0.02**
MLD (Gy)	2.5 ± 1.2	2.7 ± 1.3	2.6 ± 1.2	**<0.001**	**0.004**	**<0.001**

Mean ± SD and *p*‐values were reported. Significant *p*‐values are highlighted in bold.

Abbreviations: MLD, mean lung dose; n.s., not significant.

### 3.3 Planning times and plan complexity

The *k‐*Halcyon planning time was less than 30 min. This is compared to the estimated dedicated planning time of 180 ± 30 min (for an experienced planner) to manually create a high‐quality non‐coplanar VMAT lung SBRT plan. This estimation solely accounts for dedicated planning time and no other parts of the planning workflow such as contouring. In practice, this is not feasible as the planners frequently work on multiple plans simultaneously, must wait for physician's time for contouring, or other various checks prior to the final plan approval. Plan complexity was assessed using total MU and its derived metrics that include MF and the corresponding BOT (Table 4) as described above. There was no statistically significant difference in MU between *m‐*Halcyon and *c‐*Truebeam plans; however, MU increased for *k‐*Halcyon plans by 939 MU on average (*p* < 0.001) compared to *c‐*Truebeam plans. This corresponds to an increased MF of 0.94 (*p* < 0.001) indicating *k‐*Halcyon plans are modulated higher than both manual plans. Despite this increase of modulation, no clinically significant differences in agreement between the Eclipse TPS and the second check MU calculated dose were observed. Due to maximum dose rate restrictions (800 MU/min), BOT inherently increases by 2.86 and 1.66 min, on average, in *k‐*Halcyon and *m‐*Halcyon plans relative to *c‐*Truebeam, respectively.

**TABLE 4:** Evaluation of plan delivery metrics for 20 lung stereotactic body radiation therapy (SBRT) validation cases including original Truebeam plans

Parameter	*k*‐Halcyon	*m*‐Halcyon	*c*‐Truebeam	*k*‐Halcyon vs. *m*‐Halcyon, *p* value	*k*‐Halcyon vs. *c*‐Truebeam, *p* value	*m*‐Halcyon vs. *c*‐Truebeam, *p* value
Total MU	4076 ± 608	3126 ± 745	3137 ± 873	**<0.001**	**<0.001**	n.s.
MF	4.08 ± 0.6	3.12 ± 0.7	3.14 ± 0.9	**<0.001**	**<0.001**	n.s.
BOT (min)	5.10 ± 0.8	3.90 ± 0.9	2.24 ± 0.6	**<0.001**	**<0.001**	**<0.001**
MC second check results (%)	98.6 ± 1.9	99.9 ± 2.4	98.4 ± 2.0	n.s.	n.s.	**0.03**

Mean ± SD and *p*‐values were reported. Significant *p*‐values are highlighted in bold.

Abbreviations: BOT, beam‐on‐time; MC, Monte Carlo; MF, modulation factor; MU, monitor units; n.s., not significant.

### 3.4 LUL example case

**FIGURE 4:**
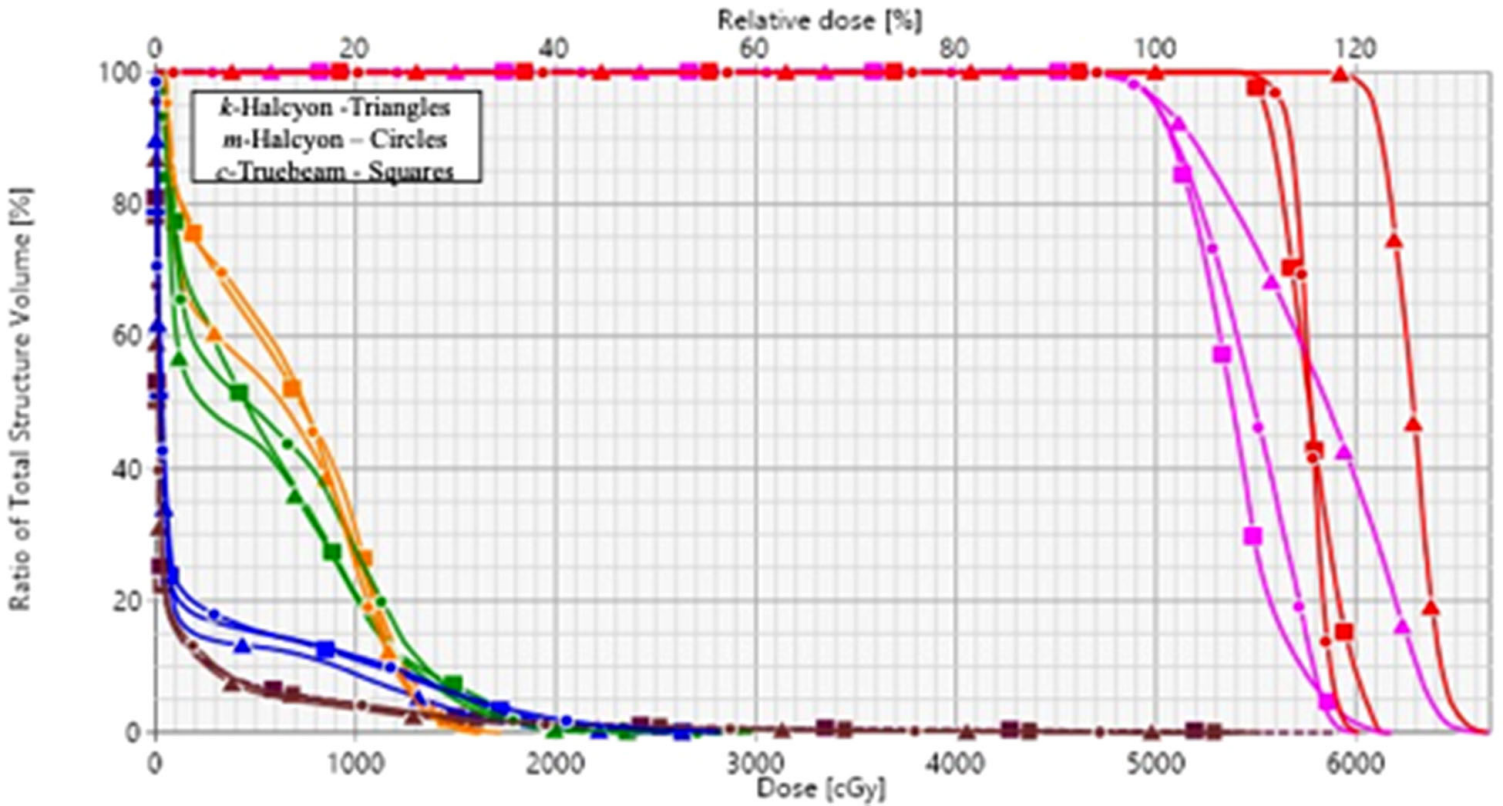
Cumulative dose–volume histogram for an example left upper lobe (LUL) patient is presented. The cumulative DVH displays selected organs‐at‐risk (OAR) and targets that include: gross tumor volume (GTV) (red), planning target volume (PTV) (pink), trachea (orange), ribs(green), esophagus (blue), and lungs‐PTV (brown) for the *k‐*Halcyon (triangles), *m‐*Halcyon (circles), and *c‐*Truebeam plan (squares). In this case, the *k‐*Halcyon plan was able to significantly increase GTV dose while maintaining similar or better OAR sparing with similar intermediate dose spillage relative to both *m‐*Halcyon and *c‐*Truebeam plan

**FIGURE 5:**
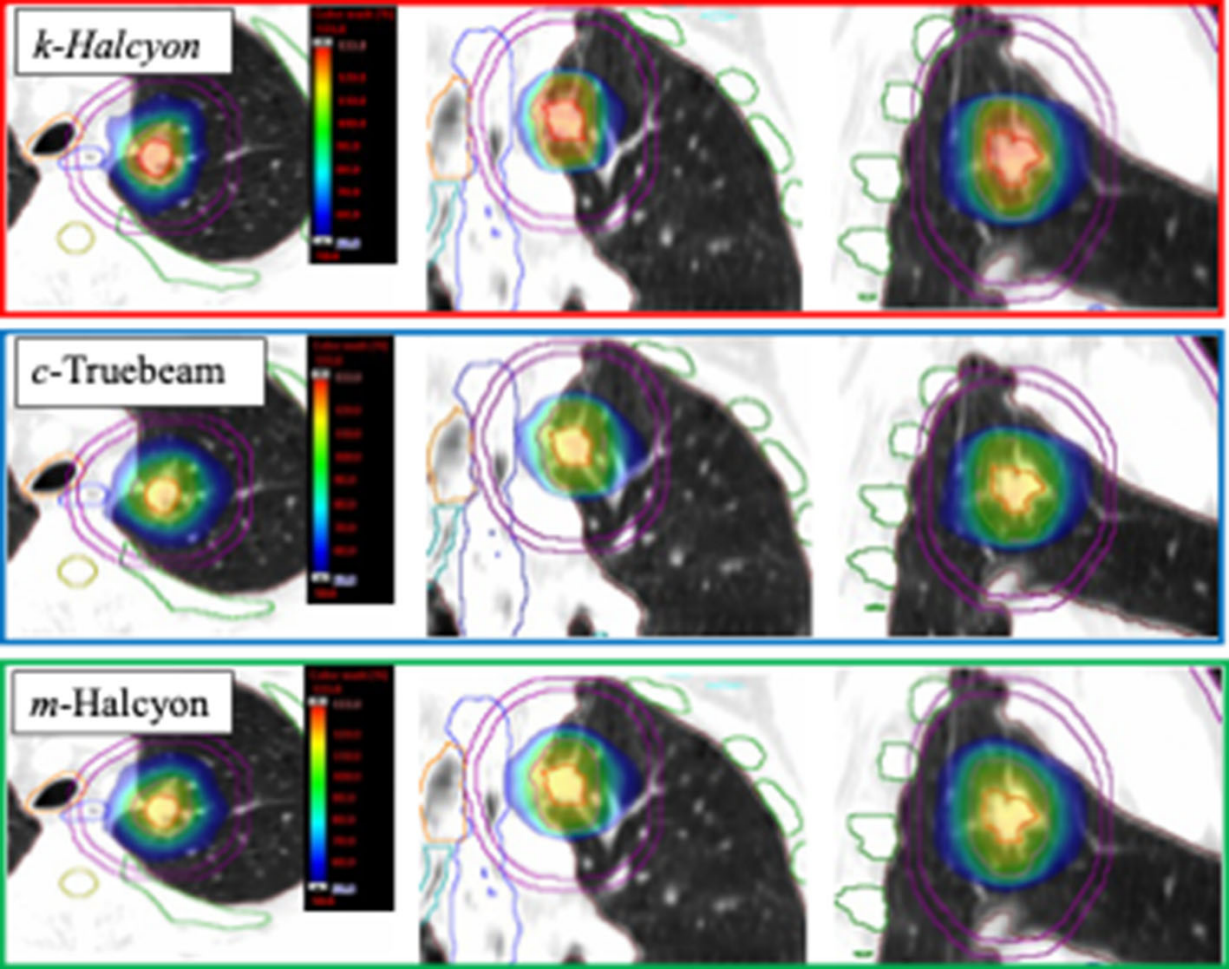
Corresponding all three views for the example left upper lobe (LUL) patient seen in Figure 4. Each view includes the same OAR and targets represented in the previously shown DVH. Note the *k‐*Halcyon plan displays a larger central hot‐spot when compared to both *c*‐Truebeam and *m‐*Halcyon plans. In all plans, tight radio‐surgical isodose color‐wash distributions are shown with the blue representing 50% prescription dose

Figures 4 and 5 present an example patient with a left upper lobe (LUL) lesion with the typical findings. The cumulative dose–volume histogram and corresponding three‐plane view through the isocenter are presented for each plan. As shown in the dose volume histogram (DVH), the *k‐*Halcyon plan (triangles) is able to escalate the GTV minimum dose when compared to both *m‐*Halcyon (circles) and *c‐*Truebeam (squares). In this case, relative to *c‐*Truebeam, GTV minimum dose was escalated by 4.6 Gy with minimal to no additional cost to OAR sparing (Figure 4). For example, the esophagus (blue) is the most proximal OAR to the target. The *k‐*Halcyon plan provided at least 1.3 Gy lower maximal esophageal dose compared to the *c‐*Truebeam plan. This dose sparing was accomplished in conjunction with a clinically significant GTV dose escalation. Similar intermediate dose spillage can be seen (Figure 5) for all three plans, with D2cm values reported to be 45% for both *k‐*Halcyon and *m‐*Halcyon plans compared to 46% in the *c‐*Truebeam plan.

## 4 DISCUSSION

This study appears to be the first to evaluate the use of a KBP model for SBRT treatment of centrally located early‐stage NSCLC patients using the ring‐mounted coplanar Halcyon Linac. While manually generated lung SBRT plans on Halcyon were dosimetrically comparable to clinically treated plans on SBRT‐dedicated Truebeam Linac, the lung SBRT KBP model (originally trained and validated using non‐coplanar Truebeam VMAT plans) was able to generate high‐quality coplanar plans on Halcyon. This was accomplished with a much shorter treatment planning time and eliminated interplanner variability. This KBP model offers a viable alternative to an SBRT‐dedicated C‐arm linac for selected lung SBRT patients. It may also be inferred that these plans were of similar quality to the Truebeam clinical plans because of the better utilization of the improved SX2 MLC on Halcyon. Additionally, dose to normal lung was significantly improved in *k‐*Halcyon compared to both *m‐*Halcyon and *c‐*Truebeam plans.

A commercially available treatment planning system RayStation (RaySearch Laboratories, Stockholm, Sweden) offers a unique feature termed “fall‐back (FB)” planning module for adaptive replanning.^38^ This module enables generation of 3D‐CRT, IMRT, or VMAT plans based on reference plans for any treatment modality using a dose‐mimicking algorithm with minimal planner time and effort. Recently, a few investigators have demonstrated that the FB planning module can convert Helical Tomotherapy plans to C‐arm linac for various sites including conventionally fractionated treatment to brain tumors, head and neck, pelvis, prostate, and lung tumors.^39^, ^40^It was reported that these FB plans were dosimetrically comparable to the original clinical plans and allowed for the fast and easy transfer of patients between treatment modalities during an unforeseen period of machine downtime.^39^, ^40^ For instance, Yuan et al.^40^ showed that FB plans would typically be generated on average for one to five fractions of the conventionally fractionated treatment course in the event of machine breakdown. Furthermore, their results suggested that an overall <1% dose variation can be achieved on target coverage and dose to OAR on FB plans. These FB plans were typically generated in 10–20 min per case so that the patient can be treated on another machine. As of now, this feature is not available in Varian Eclipse. In the case of longer machine downtime, a full re‐plan on another machine would be required, resulting in significant treatment course delay. Our KBP model could emulate the ability of the RayStation FB planning module and enable our clinic to transfer lung SBRT patients between Halcyon and Truebeam Linacs (if required) by generating a similar quality plan in less than 30 min planning time. Furthering this thought, various treatment units are not always beam matched in busy and larger clinics or all commissioned to a golden beam dataset. Therefore, utilizing a KBP model would be vital to transferring these patients between the modalities the same or next day, reducing the chance of delaying the treatment course.

There are some limitations to this study and to the Halcyon Linac. An important limitation is that, at the time of this manuscript preparation, our clinic has treated a limited number of centrally located lung SBRT patients on Halcyon. In the future, as more select lung SBRT patients are treated on Halcyon, it would be interesting and useful to include these patients in the training dataset to form a hybrid model with the clinical Truebeam SBRT plans. This may further improve the *k*‐Halcyon's model performance and may potentially create even higher quality lung SBRT plans for prospective patients. Mechanically speaking, our Truebeam Linac is equipped with a perfect pitch couch with 6‐degree of freedom (6DOF) couch corrections that allows for more accurate target localization compared to the Halcyon Linac. Additionally, for 6MV‐FFF beam, the Truebeam Linac allows for a higher dose rate of maximum up to 1400 MU/min while the Halcyon maximum achievable dose rate of 800 MU/min (increasing the BOT). This dose–rate discrepancy allows for treatments will be still clinically practical as a five‐fraction treatment scheme required relatively lower MU per fraction to be delivered the prescribed dose. Meaning, this added BOT can be offset with regard to overall treatment time using the Halcyon's “one‐step patient set‐up” capabilities as described above. However, as of now, Halcyon may not be suitable to treat lung SBRT patients with other commonly used extremely large fraction sizes (e.g., 54–60 Gy in three fractions or 30–34 Gy in one fraction)^41^, ^42^, ^43^ due to relatively longer treatment time. Future work will include investigating the feasibility of utilizing KBP models to generate lung SBRT plans with other fractionation schemes for both centrally and peripherally located lung tumors on Halcyon Linac.

## 5 CONCLUSION

This study reports on the plausibility of generating lung SBRT plans for centrally located early‐stage NSCLC patients on ring‐mounted Halcyon Linac using a previously trained and validated Truebeam KBP model. It has been demonstrated that the KBP model can be used to generate high‐quality lung SBRT plans on the Halcyon Linac that are dosimetrically equivalent or better quality when compared to manually generated Halcyon and SBRT‐dedicated Truebeam plans. This lung SBRT model is capable of quickly generating SBRT plans to support a curative SBRT treatment for centers by assuring that treatments are delivered in a safe and consistent manner potentially allowing for offline adaptive re‐planning, if needed. Additionally, the results of this study indicate that KBP models can be cross‐compatible between SBRT‐dedicated C‐arm and O‐ring linacs for lung SBRT. It is clinically useful to enable a clinic's ability to facilitate a smooth transfer of patients between treatment machines as it will ensure minimal to no treatment course disruption. This model can be shared and may provide confidence in centers equipped solely with the Halcyon Linac in the treatment of lung SBRT in the future. It may also be a great option for diverse centers with a high SBRT volume, or for patients who require an immediate SBRT.

## CONFLICT OF INTEREST

The authors declare no conflict of interest.

## AUTHOR CONTRIBUTIONS

Damodar Pokhrel and Justin Visak conceived the project. Justin Visak developed and Justin Visak and Aaron Webster validated KBP model, collected and analyzed the data. Mark E. Bernard, Mahesh Kudrimoti, Ronald C. McGarry and Marcus E. Randall provided clinical expertise and supervision of the paper. Justin Visak and Damodar Pokhrel drafted the manuscript and all co‐authors revised and approved the final manuscript.

## Data Availability

Research data are not shared.
